# Survey on Multi-Objective Task Allocation Algorithms for IoT Networks

**DOI:** 10.3390/s23010142

**Published:** 2022-12-23

**Authors:** Dominik Weikert, Christoph Steup, Sanaz Mostaghim

**Affiliations:** Faculty of Computer Science, Otto-von-Guericke University Magdeburg, 39106 Magdeburg, Germany

**Keywords:** task allocation, IoT, optimization, multi-objective, survey

## Abstract

The Internet of Things (IoT) has been an area of growing research interest for decades. Task allocation is an important problem for the optimized operation of Internet-of-Things networks. This paper provides an overview of recent research in the field of Internet-of-Things task allocation optimization. First, the task allocation problem for the IoT itself is analyzed and divided into distinct sub-problem categories, such as deployment optimization, static or dynamic optimization as well as single- or multi-objective optimization. Following that, the commonly used optimization objectives are explained. Various recent works in the field of task allocation optimization are then summarized and catalogued according to the problem categories. Finally, the paper concludes with a qualitative analysis of the categorized approaches and a description of open problems and highlights promising directions for future research.

## 1. Introduction

The Internet-of-Things (IoT) and its subset Wireless Sensor Networks (WSNs), in which all devices have a limited energy supply and use wireless-only communication, have been an area of growing research interest for decades [1]. With increasing applicability and adaption, more focus is put on the optimization of task distribution within the network, employing differing optimization goals such as the network’s lifetime, latency and reliability. Because the performance of the network regarding those metrics is highly dependent on where in the network tasks are allocated, solving the Task Allocation Problem (TAP) is a critical challenge in the IoT. Due to the inherently heterogeneous and dynamic nature of IoT networks, finding a task allocation that is optimal in terms of a single optimization goal is already challenging because of the basic NP-hard Task Allocation Problem [2]. However, with the increasing range of application and user requirements, optimizing for multiple objectives and providing multiple solutions to adjust the system in real time becomes increasingly important. With additional objectives, the already difficult problem of Task Allocation becomes even more challenging. Multi-Objective Optimization techniques and especially the concept of Pareto-Optimality and Meta-Heuristic Optimization provide solutions to these challenges.

**Figure 1:**
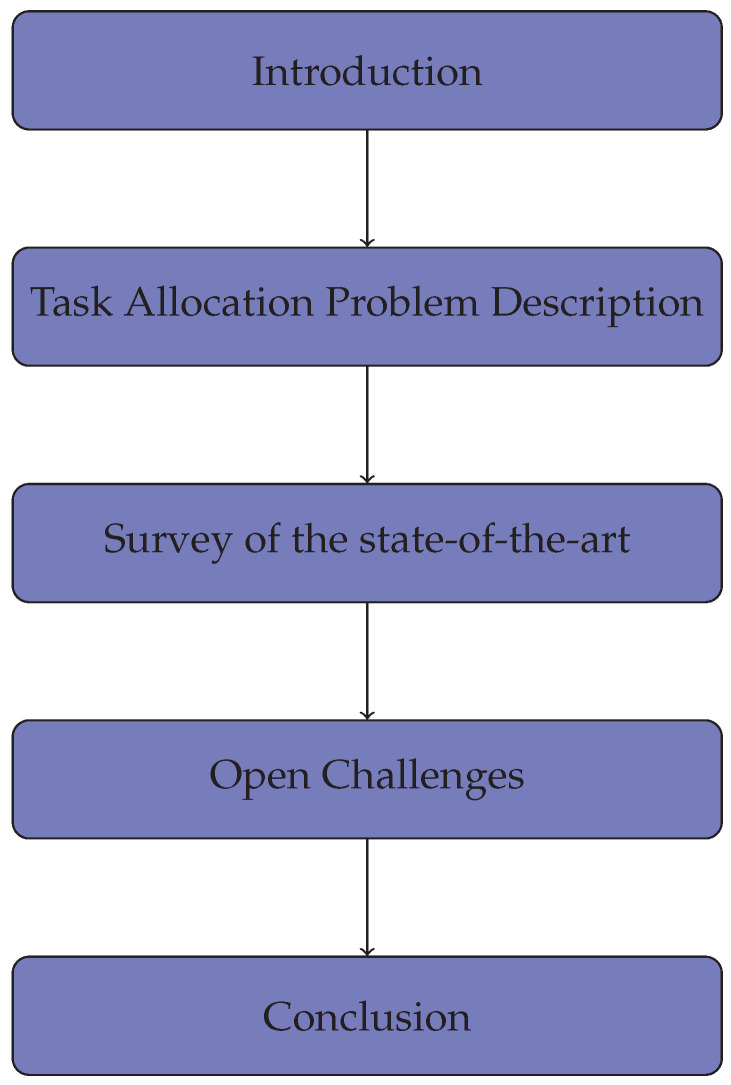
Illustrated layout of this paper.

Existing studies of the state-of-the-art for the Task Allocation Problem in IoT either focus on only a subset of the overall IoT landscape—such as crowdsensing [3,4] and WSNs [4,5]—or do not regard Multi-Objective Optimization as an important criterion to classify the surveyed approaches [3,4,5,6,7,8]. This work aimed to provide an overview of all recent work published on the TAP for the IoT, and analyze them by their adaptation of Multi-Objective Optimisation (MOO) techniques as well as their incorporation of the distinct challenges provided by IoT infrastructure. The layout of this paper is illustrated in Figure 1.

We begin with an analysis of the TAP for IoT Networks and its unique challenges. The TAP will be divided into distinct sub-categories in Section 2. Alongside the problem itself, the common metrics used to evaluate and optimize the TAP will be defined. Section 3 provides an overview of the commonly used methods to solve the TAP. With the problem and potential avenues of solving it established, the final contribution of this work is the summary of recent (i.e., mainly focusing on research published since 2019) work in this field in Section 4, and cataloging it according to the previously defined categories. Section 5 details open problems and challenges, along with promising directions for future research. The paper concludes with a summary of our findings in Section 6.

## 2. Task Allocation Problem

In this section, we will define the Task Allocation Problem for IoT and its distinct sub-problems.

### 2.1. Static Task Allocation

In the basic case of the TAP, there is a static network and a static set of tasks, i.e., neither the devices nor tasks change over time. In the state-of-the-art, graph-based representations are commonly used to describe both network structure and task set.

#### 2.1.1. Static Network Model

If there is symmetrical communication between nodes in a static network, an undirected graph is sufficient. The graph $G_{Net}=\left(N,E_{Com}\right)$ fully describes the network, with the set of graph vertices, or nodes, N, encompassing all network devices. In the remainder of this work, the terms “devices” and “nodes” will be used interchangeably. Communication links between devices are modeled by the edge set $E_{Com}$ of the graph. Each link in the network is directly represented by an edge in the graph. Each node $N_{i}\in N$ is assigned a set of attributes based on the represented device. When modeling a static network, relevant attributes are limited to the device location and its hardware. For each device, a vector $\vec{x_{i}^{N}}$ denotes its position. Relevant hardware attributes are application and device dependent, but usually include at least sensor/actuator availability $S_{i}^{N}$, processing power $p_{i}^{N}$, and memory capacity $m_{i}^{N}$. The edges of the graph are each assigned an energy consumption, $E_{ij}\left(t\right)$ which specifies the amount of energy necessary to communicate one unit of information. Additionally, a latency value $l_{ij}\left(t\right)$ represents the time necessary to transmitting data along this edge. Further edge attributes may include privacy and security specifications. A full list of the referenced node and edge attributes is given in Table 1.

#### 2.1.2. Static Task Model

Static Tasks are commonly modeled using a directed graph, which allows for the inclusion of task dependencies. A set of tasks is described by the graph $G_{Task}=\left(T,E_{Task}\right)$. The set of vertices T encompasses all tasks $T_{i}$ to be executed on the network. Weights will be assigned to each vertex to model the attributes of the represented task. An example for this is the set of task constraints $C_{i}^{T}$, used to encode the specific requirements or restrictions of task $T_{i}$. This includes a set of sensor requirements $S_{i}^{T}$ and spatial constraints $\vec{X_{i}^{T}}$ that are used to constrain the locations and nodes where the task may be executed. Sensor requirements model the specific sensors needed to fulfill the task, i.e., a temperature sensor to monitor heat in a system or a camera for a surveillance-based task. The spatial constraints $\vec{X_{i}^{T}}$ complement this by specifying the exact location where each task has to be executed. This may be highly specific, restricting the task location to a single room within a building, or more expansive regions such as a city block or street for traffic monitoring. However, some tasks, especially those that only process or collect data, may not have any such restrictions at all and may be allowed to be assigned to any node within the network. Beyond the sensor and spatial constraints, there may be minimum hardware requirements, e.g., concerning processing power $q_{i}^{T}$ and memory capacity $m_{i}^{T}$. Task dependencies are modeled by the task graph’s set of edges $E_{Task}$. Each edge $e_{ij}$ denotes a directional dependency between tasks $T_{i}$ and $T_{j}$. Edge weights may be used to model characteristics of the dependency, such as the Communication Costs ($c_{ij}$) between tasks.

**Figure 2:**
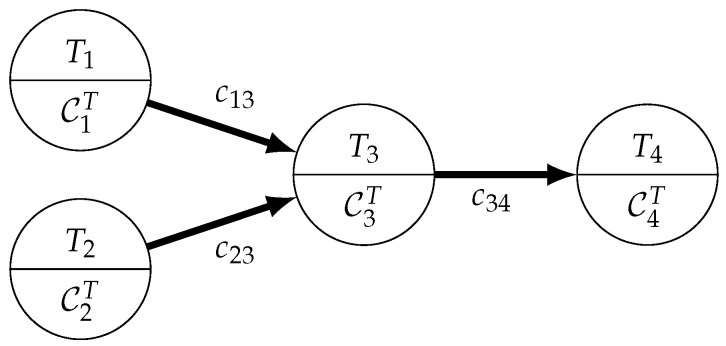
Basic task graph example.

An example task graph with two sensing tasks producing data ($T_{1}$ and $T_{2}$), one processing task ($T_{3}$) accumulating the information from both sensing tasks and one task ($T_{4}$) acting as a data sink is shown in the Directed Acyclic Graph (DAG) visible in Figure 2.

While solving the TAP is already an NP-hard challenge by itself [2], the TAP for the IoT presents an even more complex problem due to the following dynamic elements it presents.

### 2.2. Dynamic Task Allocation

Real-world applications inherently include dynamic elements. As such, an extension of the models described previously is needed to allow for such elements at both the task and network levels. Network dynamics mainly have two causes: Movement of the network’s devices, and the addition or removal of devices from the network.

#### 2.2.1. Device Mobility

Device mobility can be included in the network model by adopting a time-based component *t* into the network graph $G_{Net}\left(t\right)=\left(N\left(t\right),E_{Com}\left(t\right)\right)$. Within the sets of nodes and edges of the graph, this time-based variable modifies the node position $\vec{x_{i}}\left(t\right)$, which in turn affects the set of edges $E_{Com}\left(t\right)$ as communication links between nodes may both be lost and newly formed as the nodes physically move. Furthermore, the observance of task spatial constraints $\vec{X}$ may be affected. Device mobility presents an additional challenge for solving the TAP because the optimal allocation may keep changing, and thus very limited time may be available for the optimization process, which needs to be adapted for every movement of a node. Because optimal allocations have to be ready for any new node positions, prediction of movement becomes a key challenge in a mobile network.

#### 2.2.2. Device Failures

The removal of devices is introduced through failures of the devices themselves or their components. Such failures may not be permanent, such as during short-term communication outages or sensor failures. Permanent failures could be caused by the exhaustion of device energy $q_{i}$ or a device leaving the network. To model this, the hardware availability $S_{i}^{N}$ of node $N_{i}$ is adapted to a function mapping availability of each hardware component *s* for each device, as shown in Equation (1).

(1) $$\begin{array}{cc} S_{i}^{N}\left(s,t\right) & =\left\{\begin{array}{cc} 1, & \mathrm{component}s\mathrm{is}\mathrm{available}\mathrm{on}N_{i}\mathrm{at}\mathrm{time}t,\\ 0, & \mathrm{otherwise} \end{array}\right. \end{array}$$

To model the failure of an entire device, the representing node can simply be removed from the network graph. Alternatively, a component representing the entire device can be included in $S_{i}^{N}\left(t\right)$ to keep the ordering and indexing of nodes consistent throughout node failures and additions.

Device failures present additional challenges for solving the TAP due to their unpredictability. Any allocation may be invalidated at any time due to the failure of a node or component. Unlike mobility, such failures are impossible to predict and, with the exclusion of failures due to an exhausted power supply, there is no way to know whether the error is transient or permanent.

### 2.3. Task Allocation Problem

Formally, the TAP is a search problem of an injective function a:T→N(t), which assigns each vertex in the task graph $T_{i}$ to a vertex $N_{j}$ of the network at the time *t* according to the Position Constraint ($S_{i}$) of the tasks ${\vec{x}}_{j}\left(t\right)\in S_{i}$. Additionally, the node $N_{j}$ executing the task $T_{i}$ needs to be connected through communication edges $e_{jl}\in E_{com}$ to all nodes $N_{l}=a\left(T_{k}\right)$ executing directly connected tasks $e_{ik}\in E_{Tasks}$. Since nodes can dynamically fail, the goal is to find a series of valid allocations $A=\left(a_{0},a_{1},\cdots ,a_{n}\right)$ with associated start times $t_{i}^{start}$ and end times $t_{i}^{end}$, for i=0,⋯,n. The allocation denoted as $a_{n}$ is the last allocation possible. After this allocation, the remaining network is not able to continue executing the global task *T* anymore, which ends the lifetime of the network $t_{n}^{end}$. The goal is then to find such a series of allocations A that optimize certain network metrics during the lifetime of the network.

### 2.4. Task Allocation Optimization Metrics

An important part of solving the TAP are the metrics by which to optimize. This section will define the most commonly found metrics in the literature in accordance with the network and previously given problem definition.

#### 2.4.1. Network Lifetime

Network Lifetime is defined as the difference between the latest end time $t_{n}^{end}$ of the last valid allocation $a_{n}$ and the start time of the first valid allocation $t_{0}^{start}$ [9]:

(2) $$NL\left(A\right)=t_{n}^{end}-t_{0}^{start}$$

Network Lifetime measures the total time the network as a whole and is capable of carrying out the assigned tasks. As such, it becomes important for networks wherein replacing the batteries may be expensive or completely impossible. In mixed networks, optimizing for network lifetime may put more strain on devices with large or inexhaustible power supplies at the cost of overall efficiency.

#### 2.4.2. Energy Consumption

The Energy Consumption of an allocation is the sum of all energy dissipated from all nodes while the allocation is currently applied to the network, as displayed in Equation (3) [10].

(3) $$E\left(a_{n}\right)=\sum_{N_{i}\in N}\left(q_{i}\left(t_{n}^{start}\right)-q_{i}\left(t_{n}^{end}\right)\right)$$

Consequently, the energy consumption of an allocation series, E(A), is the sum of all the energy consumption of all allocations in that series:

(4) $$E\left(A\right)=\sum_{a_{n}\in A}E\left(a_{n}\right)$$

Energy consumption usually becomes relevant in scenarios where the absolute extension of a network lifetime is less important, e.g., with renewable energy sources or reliable power supplies.

#### 2.4.3. Latency

The Latency $l_{ij}$ for each connected pair of nodes i,j is given by the edge weights $l_{ij}$ connecting them. The latency between dependent tasks $T_{k}$ and $T_{l}$ can thus be defined as the sum of all latencies along the path $P_{kl}\left(a_{t}\right)$ plus the node latencies $ml_{k}$. The latency $L(a_{t})$ of the allocation $a_{t}$ is defined as the maximum latency of all connected tasks. The Latency of an allocation series A is the maximum latency of any allocation of the series [9]:

(5) $$L\left(A\right)=\max_{a_{t}\in A}\left[\max_{T_{k},T_{l}\in V_{Tasks}}\sum_{e_{ij}\in P_{kl}\left(a_{t}\right)}l_{ij}+l_{i}\right]$$

Latency in this way measures the maximum time that the system may take to respond to a request or to perform an overall task. As such, it is important in customer-focused applications or time-critical scenarios, where the response time of the system is an important factor. Latency is also often used as a constraint by employing deadlines, enforcing the system to discard any allocations which cannot observe a certain minimum latency value, but not optimizing the latency beyond said constraint.

#### 2.4.4. Availability

Availability is defined based on the error indicator function *e*, see Equation (6), as the fraction of time when the network Net is fulfilling the task *T* (i.e., the network is error-free e(A,t)=0), divided by Network Lifetime (NL(A)) [9]:

(6) $$\begin{array}{cc} e(A,t) & =\left\{\begin{array}{cc} 0, & a_{i}\mathrm{is}\mathrm{valid},t_{i}^{start}\leq t<t_{i}^{end}\\ 1, & \mathrm{otherwise} \end{array}\right. \end{array}$$

(7) $$\begin{array}{cc} A(A) & =1-\frac{\int_{t_{0}^{start}}^{t_{n}^{end}}e\left(A,t\right)dt}{NL(A)} \end{array}$$

Availability measures the overall uptime of the system. It becomes important in networks with regular outages, where error correction is a focus, or systems that need to have as much fault tolerance as possible because the downtime of the system could have serious consequences. Beyond these applications, it is also critical to provide a good user experience.

#### 2.4.5. Reliability

Reliability (*R*) is defined as the time of the first fault $t_{A}^{error}=\underset{t}{\mathrm{argmin}}\left(e(A,t)=1\right)$ leading to the network not being able to execute the global task *T* divided by the Network Lifetime [11].

(8) $$R\left(A\right)=\frac{t_{A}^{error}}{NL(A)}$$

As such, reliability measures the fraction of time during which the network operated before an error disturbed the execution of any task. It therefore is a relevant metric for any security-critical or medical application.

## 3. Methodologies for the Task Allocation Problem

This section will briefly explain the most commonly found algorithms in the state of the art to facilitate our understanding of the latter analysis of recent works in this field.

### 3.1. Multi-Objective Optimization

Given the example metrics of the previous section, it might be desirable to optimize both Latency and Network Lifetime, which typically conflict because Latency is improved by bringing tasks close together, optimally on a single node, which drains the battery of these nodes faster, impacting the Network Lifetime. Such problems are called Multi-Objective Problems (MOPs) on objectives $f_{i}$, formally defined as seen in Equation (9).

(9) $$\begin{array}{cc} \; & \min\left(f_{1}\left(\vec{x}\right),f_{2}\left(\vec{x}\right),\cdots ,f_{m}\left(\vec{x}\right)\right)\\ \; & s.t.\vec{x}\in S\\ \; & \;\vec{g}\left(\vec{x}\right)\leq 0,\vec{h}\left(\vec{x}\right)=0 \end{array}$$

Such a problem consists of *m* objective functions, which have to be optimized, i.e., minimized or maximized. Each of these objective functions maps from the search space S, which contains all possible solutions $\vec{x}\in S$ to the problem, to an objective space of dimension $R^{m}$ by assigning each solution a certain fitness vector. Additionally, the search space may be constrained, which is formally expressed by inequality constraints $\vec{g}$ or equality constraints $\vec{h}$. Any solution $\vec{x}\in S$ has to satisfy these constraints. For simplification, the remainder of this work assumes that all objective functions have to be minimized, which does not impact generality because maximization objectives can be transformed into minimization objectives by $\max\left(f_{i}\right)\equiv \min\left(-f_{i}\right)$. In case of metrics that are to be maximized, such as Network Lifetime, the metric will be inverted accordingly to achieve a minimization problem. The nature of the search space is dependent on the problem. Often, it is a subspace of R for real-valued problems. However, binary or integer-valued problems exist. In this work, solutions are allocations of tasks to nodes in the network. As such, the dimensionality *n* of the search space is equal to the number of nodes present, and solutions are encoded in the subspace of $I^{n}$. In scenarios where the assignments of tasks to multiple nodes are possible or desired, e.g., for the sake of redundancy, the search space becomes exponentially bigger and solutions are encoded by assigning tasks to sets of integers instead of a single integer.

The main challenge in any MOP is the ranking of solutions against one another. Since multiple conflicting objectives exist, finding a single optimal solution is typically impossible. To combat this, the concept of Pareto-Optimality is used. Generally, Pareto-Optimality is achieved in a solution if no objective can be improved without devaluing another. To rank solutions against one another, a domination criterion called Pareto-Dominance may be applied. Both concepts are defined as follows in Definitions 1 and 2.

**Definition 1**  (Pareto-Dominance). *A solution $\vec{x}\in S$ dominates another solution $\vec{y}\in S$, if $\vec{f}\left(\vec{x}\right)$ is at least equal to $\vec{f}\left(\vec{y}\right)$ in all objectives, and there exists at least one objective where $\vec{f}\left(\vec{x}\right)$ outperforms $\vec{f}\left(\vec{y}\right)$, see Equation (10).*

(10) $$x≻y\Leftrightarrow \vec{f}\left(x\right)\geq \vec{f}\left(y\right)\vee \exists f_{i},f_{i}\left(x\right)>f_{i}\left(y\right)$$

**Definition 2**  (Pareto-Optimality). *A solution $\vec{x}\in S$ is Pareto-Optimal, iff it is not dominated by any other solution in the search space. The set of all Pareto-Optimal solutions is called the Pareto-Front PF, see Equation (11).*

(11) x∈PF⇔∄y,y≻x

### 3.2. Base TAP Algorithm

Before the different methods are described, this section will give a short description of an example algorithm to solve the full TAP, which can be used in combination with any of the algorithms described below. The algorithm is described in Algorithm 1. The minimal input for any optimization algorithm includes the network and task graphs, along with a time value which determines how much time will be looked ahead in any predictive models, such as mobility models. An initial allocation can be created and deployed to the network before full optimization begins. Then, until the current time reaches the prediction horizon determined by adding the look-ahead time δt to the current time, the optimization algorithm is executed to generate at least one new allocation $a_{new}$. Using a predictive mobility model, the mobility of the nodes can be taken into account and the optimization performed on the predicted future network state. If a failure is detected at any time during this loop, any recovery mechanisms of the optimization algorithm should be carried out to quickly generate a valid allocation. If no such mechanism exists, the availability of the network will be impacted accordingly until a new allocation is generated. Finally, when the current prediction horizon is reached, the new solution may be deployed to the network and the previous steps are repeated until the network is no longer operational. Any of the algorithms described in the following would replace lines 2 and 8 with their specific optimization procedure. Specific solutions providing a recovery mechanism for node failures could implement this in place of line 11.

### 3.3. Metaheuristics

Multi-Objective optimization problems are often solved with Metaheuristic Optimization Algorithms, which are commonly applied to problems which cannot be solved by exact or analytical means, such as NP-hard problems. Metaheuristics usually make little assumptions about the problem to be solved, and provide no guarantee of optimality regarding the obtained solutions. As such, they usually provide a tradeoff between solution quality and the required computation time. For the scope of this work and the Task Allocation Problem, the most notable metaheuristics are Evolutionary Algorithms (EAs) [12,13], Ant-Colony-Optimization (ACO) [13,14,15] and Particle Swarm Optimization (PSO) [13,16]. As such, these approaches will be shortly explained in the following subsections.
**Algorithm 1:** Base procedure for solving the TAP
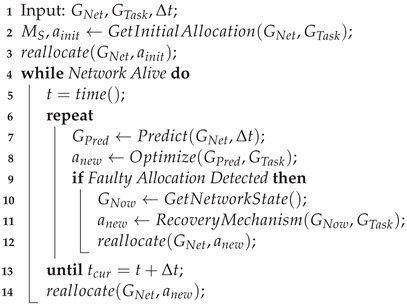


#### 3.3.1. Evolutionary Algorithms

EAs are, as the name suggests, inspired by the principle of biological evolution. They operate on a set, or population, of possible solutions a,∈P and as such are part of the population-based metaheuristics. The initial population is filled by randomly generating solutions. Afterwards, the set of solutions is gradually improved by applying the principles of natural evolution through selection, genetic recombination and mutation. The basic procedure of a common type of EA (μ+λ EA) is outlined in Algorithm 2: The first step is to initialize the population, e.g., with μ randomly created individuals. Then, each individual is evaluated according to the objective functions $\vec{f}={\left(f_{0},\cdots ,f_{i},\cdots ,f_{m}\right)}^{T}$ of the given problem. According to the calculated fitness, the first selection ${\mathrm{select}}_{0}$ is carried out to determine λ individuals, which will “reproduce”. These individuals are then recombined (crossover), and mutations are applied to their generated “offspring” (mutate). Afterwards, the newly created individuals are evaluated. The last step in the EA loop is to select a new population of μ “survivors” from the original population and the offspring (${\mathrm{select}}_{1}$). ${\mathrm{select}}_{0}$ and ${\mathrm{select}}_{1}$ may be the same operation. Typical termination criteria involve a maximum number of generations or reaching a certain fitness threshold.

#### 3.3.2. Ant Colony Optimization

ACO is another nature-inspired Metaheuristic. Based on the foraging behavior of an ant colony, artificial ants move throughout the search space, leaving artificial pheromones based on the quality of the solutions. The movement of the ants is based upon the pheromone concentration in a probabilistic fashion, causing more ants to take the paths that lead to better solutions. Due to the path-based optimization, ACO is well suited to problems involving graphs and routing. In a graph-based problem, any ant *k* travels along the edges to visit different nodes. Starting on the root node i=0, each ant *k* decides where to go next, based on the probability $p_{ij}^{k}$, given by Equation (14). $N(s^{p})$ denotes the set of feasible edges, e.g., all the reachable edges the ant has not visited yet, to prevent the ants from going in circles. *Q* is a constant, and $L_{k}$ the length of path taken by ant *k*. This decision is repeated until the target node is visited. The parameters α and β are used to scale the impact of the pheromone information $\tau_{ij}$ versus the heuristic information $\eta_{ij}$, which is dependent on the optimization problem. After a full solution has been created by an ant, the pheromone values $\tau_{ij}$ of all edges $e_{ij}$ are updated according to Equations (12) and () [14].

(12) $$\begin{array}{cc} \tau_{ij} & =\left(1-\rho \right)\cdot \tau_{ij}+\sum_{k=1}^{m}\Delta \tau_{ij}^{k} \end{array}$$

(13) $$\begin{array}{cc} \Delta \tau_{ij}^{k} & =\left\{\begin{array}{cc} Q/L_{k}, & \mathrm{if}\mathrm{ant}k\mathrm{used}\mathrm{edge}(i,j)\\ 0, & \mathrm{otherwise} \end{array}\right. \end{array}$$

(14) $$\begin{array}{cc} p_{ij}^{k} & =\left\{\begin{array}{cc} \frac{\tau_{ij}^{\alpha }\eta_{ij}^{\beta }}{\sum_{c_{ij}}\in N\left(s^{p}\right)}, & \mathrm{if}c_{\mathrm{ij}}\in N\left(s^{p}\right)\\ 0, & \mathrm{otherwise} \end{array}\right. \end{array}$$

**Algorithm 2:** Base μ+λ Evolutionary Algorithm

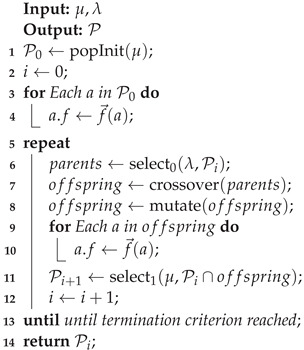



#### 3.3.3. Particle Swarm Optimization

PSO is based upon the movement of natural swarms, such as flocks of birds or school of fish. A population of so-called particles ${\vec{x}}_{i}\in P$ moves within the search space S according to simple rules: to mimic the sharing of information, each particle is attracted to the best solution that the swarm has found so far (usually called the global best). Additional attraction is experienced towards the best solution that each particle itself has found so far (referred to as the local best). At each time step *t* of the algorithm, a particle *i* occupies the position ${\vec{x}}_{i}\left(t\right)$ and moves with the velocity ${\vec{v}}_{i}\left(t\right)$. Each particle also retains a memory of its local best ${\vec{x}}_{bi}\left(t\right)$ and the global best ${\vec{x}}_{g}\left(t\right)$. The velocity and position of each particle is then updated by calculating the difference between its current position and the local and global bests according to Equations (15) and (16).

(15) $$\begin{array}{cc} {\vec{v}}_{i}\left(t+1\right) & =\omega {\vec{v}}_{i}\left(t\right)+c_{1}{\vec{r}}_{1}⊗{\vec{v}}_{i_{c}}\left(t\right)+c_{2}\vec{r_{2}}⊗{\vec{v}}_{i_{s}}\left(t\right) \end{array}$$

(16) $$\begin{array}{cc} {\vec{x}}_{i}\left(t+1\right) & ={\vec{x}}_{i}\left(t\right)+{\vec{v}}_{i}\left(t+1\right). \end{array}$$

Using the parameters $c_{1}$ and $c_{2}$, the attraction towards the local and global bests can be scaled. The random vectors $\vec{r_{1}}$ and $\vec{r_{2}}$ are used to induce additional randomness in the movement of the particles, which improves the exploration of the search space, and are applied to the velocities using element-wise multiplication ⊗. The inertia weight ω determines the influence of the previous velocity, which further increases exploration.

### 3.4. Collaborative and Antagonistic Game Theory-Based Algorithms

This section details algorithms based on game theory. Game theory applies mathematical models to the strategic behavior of agents which attempt to achieve a certain goal. This can be in an antagonistic fashion, e.g., in zero-sum games, or cooperatively where all agents attempt to achieve the best overall possible outcome. For task allocation, each node can be seen as an agent, and they may cooperate or compete over the assignment of the individual tasks.

#### 3.4.1. Consensus Algorithms

Consensus algorithms are a type of algorithm often used to ensure reliability in distributed systems. By requiring participants to agree on a value, or in the case of this work, a task allocation, single points of failure can be eliminated. An often used approach is based on message passing, in which participants broadcast their observed or desired value, and through several rounds of such exchanges and adaptations, a majority can be found. This can be used to, e.g., select a leader for a cluster of nodes, which is then mainly responsible for accepting tasks on behalf of all nodes in its cluster and clustered nodes will always accept the tasks assigned to them by the leader. This clustering approach reduces the search space and makes solving the task allocation problem easier.

#### 3.4.2. Auction Algorithms

Auction algorithms are another iterative procedure to solve combinatorial optimization problems, especially the assignment problem. By associating the objects—or in the case of this work, tasks—to be assigned with a value and a price, several rounds of bidding can take place during which participating agents (nodes) seek to maximize their profit. Bidding continues until all participants are satisfied with their assigned object. For any node *i*, this is the case if there is no other task *j* which is more profitable for it, e.g., the difference between the price $p_{j}$ and the benefit $b_{ij}$ of assigning the task is maximized, as shown in Equation (17).

(17) $$\begin{array}{c} b_{ij}-p_{j}=\max_{j=1,\cdots ,n}\left\{b_{ij}-p_{j}\right\} \end{array}$$

As long as there are participants that are not satisfied, iterative bidding is carried out. During this process, a non-satisfied node $n_{i}$ is selected, and exchanges its assigned task with another node whose task $t_{j}$ offers a maximum value. The price of task $t_{j}$ is then increased so that $n_{i}$ no longer prefers it over the second-best task, e.g., $p_{j}=p_{j}+\gamma$ where γ denotes the difference in value between the best and second best tasks. As in real-world auctions, this process raises task prices iteratively. The process runs until all nodes are satisfied with their assigned task.

#### 3.4.3. Stackelberg Game

In the Stackelberg Game, participants are divided into a leader and followers. The leader acts first and is aware of all possible reactions of the followers. With this information, the leader can maximize its profits by anticipating the follower actions, and followers can maximize their profits by employing the observed results of the leader.

### 3.5. Reinforcement Learning

Reinforcement Learning (RL) algorithms have wide applicability and are thus used in a variety of fields. The purpose of reinforcement learning is for an actor, called an agent, to learn a strategy π, also called a policy, which maximizes a user-defined “reward function” *Q*. Over the course of the learning process, the algorithm takes actions *k* that change the state *s* of the environment and gains a reward based on the quality of the action at that state Q(s,k). Over time, the agent is supposed to learn a policy that maximizes the rewards.

A widespread version of reinforcement learning is Q-learning [17,18]. In Q-learning, an agent attempts to learn an optimal policy by iteratively taking actions and observing the given reward. As such, for Q-learning, neither the expected rewards nor the state transitions need to be known beforehand. Equation (18) describes the mathematical process of updating the Q values for a policy π one step at a time: given a state–action pair (s,k), the Q-value for this pair according to policy π is given by the immediate reward $R_{t}$ of taking action *k* while in the state *s* at time-step *t*, and then following the policy π afterwards. By computing the error between this value and the original estimated Q value, the Q values are iteratively updated. α is used as a learning rate factor, to control how quickly new experiences change the estimated Q value.

(18) $$\begin{array}{c} Q_{\pi }\left(s_{t},k_{t}\right)=Q\left(s_{t},k_{t}\right)+\alpha \left(R_{t}+\gamma \max_{k}Q_{\pi }\left(s_{t+1},k\right)-Q\left(s_{t},k_{t}\right)\right) \end{array}$$

#### Deep Reinforcement Learning

Deep Reinforcement Learning is the intersection between Reinforcement Learning and Deep Learning (DL). In Deep RL algorithms, the manual mapping of the state space is usually replaced by a deep neural network, which allows for the incorporation of larger state spaces and input data.

A modified variant of Q-learning incorporating Deep Learning (DL), known as Deep Q-learning, replaces the large table that results from mapping each state–action pair (s,k) to a Q-value with a deep neural network. This network maps input states to pairs of actions and their estimated Q-values. This is beneficial for any scenario with a large search space, as a table mapping all possible state–action pairs quickly becomes prohibitively large.

### 3.6. Other Methods

This section describes approaches used in the state-of-the-art that do not fit in any of the above categories. As they are rarely used in the state of the art, they will not be described in detail. For further information on these types of algorithms, the reader is referred to the original papers.

#### 3.6.1. Linear Programming

Linear Programming (LP) is a mathematical technique for solving problems wherein the objective function is linear. As such, they do not deal with Multi-Objective Optimization as described in Section 3.1. However, methods based on LP can usually guarantee finding an optimal solution, which the methods described above cannot. Any LP problem is described by a linear function to be solved and several constraints that need to be observed. Mixed-Integer Linear Programming (MIP) is an adaptation of LP that includes non-discrete variables, such as Boolean values.

#### 3.6.2. Hill Climbing

Hill climbing is an optimization technique in which small changes are applied to an existing solution in order to incrementally improve it. If such a small change would improve the solutions, it is applied, otherwise it is discarded. By repeating this step, the solution can be optimized over time.

## 4. State of the Art on the Task Allocation Problem for IoT

There has been a variety of work on the various categories defined above. This section will detail these works, and provide a top-level overview of which approaches have been applied to the problem or parts of it.

### 4.1. Comparison Criteria

To facilitate the comparison of a vast variety of works in this field, comparison criteria based upon the problem subcategories need to be defined. These are directly based upon the categories defined in Section 2. Works will be categorized based upon their suitability to deal with the different criteria described below.

#### 4.1.1. Node Failures

The first comparison criterion pertains to the node failures described in Section 2.2.2. There are long-term failures, e.g., due to a drained battery, and short-term failures. One way to deal with failures would be to carry out a complete re-planning of the task allocation. However, this would incur potentially prohibitive time and resource drains. As such, this work considers approaches that use this approach as not suited for node failures. Approaches capable of quickly adjusting to long-term failures but not short term-failures (or vice versa) are judged as partially suited. Only approaches capable of dealing with both long-term and short-term failures are judged as fully suited to deal with this criterion.

#### 4.1.2. Node Mobility

The second criterion upon which the evaluated methods will be judged is the challenge of node mobility, as described in Section 2.2.1. Again, there exist different ways to handle mobile nodes. Complete re-planning is not feasible at all, as it would have to be performed constantly. Another approach is to see mobility-induced failures of allocations, e.g., due to connection loss or violation of spatial constraints, as node failures and deal with them similarly. Works that use an approach such as this are deemed partially suited for dealing with node mobility. However, mobility is usually not a completely random phenomenon. As such, a viable approach is to create models upon which to analyze or predict the node movement for a specific application. With a prediction mechanism, task allocations can be adapted and optimized pre-emptively, resulting in less or no uptime loss for the overall system. Methods incorporating such an approach are considered as fully suited for this challenge.

#### 4.1.3. Multi-Objective Optimization

The final comparison criterion pertains to the nature of the optimization itself. Section 2 detailed the metrics of interest to the optimization. While it is unlikely that all of them are relevant to a specific application, often multiple objectives are relevant. As such, this work will also analyze the presented approaches based upon their approach to optimizing multiple objectives simultaneously. Works that only optimize a single objective are judged as not suited at all to MOO. One way to deal with a multi-objective approach is to combine the different objectives in a weighted-sum fashion, which will be deemed partially suitable for MOO. Only approaches using a full MOO algorithm and optimizing for all metrics described in Section 2.4 will be categorized as fully suitable. Algorithms are considered a full MOO algorithm if they use the concept of Pareto-Dominance as defined in Definition 1 to rank and simultaneously optimize conflicting objectives.

### 4.2. Comparison of the State of the Art

The following subsections will analyze the state of the art for Task Allocation Problems in the IoT according to the categories defined above. A complete overview of all approaches analyzed and their respective performance in the categories is provided in Table 2. Full suitability is indicated by a+, while partial suitability is indicated by a *o*, and an unsuitable approach is designated by a−. An additional column in the table indicates the evaluated network metrics and any special observations or features of the work. In addition to the overview table, the following sections will provide the reasoning for the categorization for each work in more detail. With the goal of revealing the strengths and weaknesses of the different methods as presented in Section 3, the approaches have been divided into the same groups and will be analyzed together.

### 4.3. Metaheuristic Approaches

This section will analyze the works that fall into the category of Metaheuristics in terms of the categories defined in Section 3, beginning with evolutionary algorithms described in Section 3.3.1.

Khalil et al. [19] proposed an EA-based approach to optimizing energy consumption. As such, they considered the failure of nodes due to drained battery, but did not incorporate short-term failures. The mobility of nodes is not considered at all. They propose different fitness evaluations, prioritizing either energy consumption or node longevity, but do not include any MOO approach. Khalil et al. [20] also proposed a revised approach to optimize the energy consumption and maximize network lifetime. Their network model in terms of failures and mobility does not change, however, resulting in the same judgments. The work also does not incorporate any MOO.

Tao and Song proposed an EA-based approach [21] to optimize the allocation of tasks in mobile crowdsensing applications. No failures in the participating devices are considered, but mobility is inherently present in the problem. However, the proposed approach actively pre-plans the paths for each device, and devices are only assigned a tracking task if they follow the proposed path, instead of optimizing around nodes freely moving within the network. As such, we judge the approach as partially suitable for mobile environments. In terms of MOO criteria, the approach optimizes the accuracy of the measurements, as well as the distance traveled for each node, which can be considered a type of energy consumption. However, the distance traveled is modeled as a constrained instead of a true independent objective, resulting in a non-suitable judgment.

Sahni et al. [22] proposed another EA-based approach to optimize the energy dissipation of executing a set of tasks in IoT networks. However, they only considered static networks and did not incorporate any mechanisms to deal with node failures or mobility, and only optimized for a single objective.

A Biogeography-based optimization (BBO) algorithm was proposed by Liu et al. [23]. BBO algorithms are a type of EA which is inspired by the migration of different species between habitats. This approach tries to minimize the unavailability level of the tasks, but does not actively mitigate when failures occur. The objectives latency and availability are evaluated as well as a balanced resource wastage, but do not use the concept of Pareto-Optimality to do so, instead using a weighted-sum approach to combine objectives into one objective function.

Weikert et al. [9,11,24,25] proposed a group of EAs suited to deal with the challenges of task allocation in mobile and failure-prone networks. Their latest work [9] combines these approaches and improves upon the weaknesses of the earlier versions. Their work also incorporates a true MOO approach, optimizing network lifetime, latency and availability. However, their algorithm does not allow for redundant allocations and does not optimize for reliability at all.

The next set of metaheuristic algorithms belongs to the group of PSO algorithms as described in Section 3.3.3, another widespread method to solve the TAP.

In [26], Guo et al. developed a PSO-based optimization algorithm with a backup mechanism to achieve fault tolerance and improve the energy consumption, task execution time, energy balance and reliability. However, they combine the objectives into a weighted-sum, creating a partially MOO-capable solution. They also do not consider node mobility.

Yang et al. [27] modified a Binary PSO algorithm to optimize task allocation in terms of execution time, energy consumption and network lifetime in a weighted-sum fashion. The optimization is carried out in a static network, not considering the node failures or mobility, resulting in unfeasibility for scenarios involving these challenges.

Another PSO-based approach was developed by Wang et al. [28]. This approach incorporates some concepts of EAs into the particle optimizer in order to optimize latency. A thorough study of the impacts of the PSO parameters was carried out, but the network considered was fully static, neither incorporating failures nor mobility.

Baniabdelghany et al. [29] proposed a method to optimize the energy consumption, latency, and reliability of executing a set of tasks on a static network. This approach used a weighted-sum strategy to combine the multiple objectives into one fitness function.

In the area of network security, Sun et al. [30] proposed a task allocation mechanism based on Binary PSO to determine nodes to employ in the detection of attacks on the network in order to improve the network lifetime, reduce energy consumption and balance the network load. This work also employs the concept of Pareto-Optimality for these three objectives. However, only static networks are considered.

Zhang et al. [31] developed another PSO-based method to obtain reliable and energy-efficient task allocations in WSNs. Energy consumption is formulated as the only objective, while constraints are designed for reliability and task execution time. This work also only considered static networks and neglected the possibility of node failures and mobility.

Olmsted and Masri [32] proposed a method to dynamically optimize the task allocation process in fog environments. They combined several non-specified objective functions into a single function using the weighted-sum method. While this approach accounts for dynamically accepting new tasks during runtime, it does not consider the failure or movement of nodes in the established network.

Niu et al. [33] designed a modified PSO algorithm to optimize the computing delay, i.e., the latency of task allocations. Their approach only considers this single objective, and their system model neither incorporates mobility nor node failures.

In [34], Ren et al. investigated a combined PSO and Simulated Annealing approach to minimize the execution cost, latency and energy consumption of task allocations via a weighted-sum fitness function. However, their approach accounts for neither failures nor mobility.

The final two works in the category of metaheuristics are ACO algorithms as detailed in Section 3.3.2. Xu and Zhou [35] proposed an ACO-based approach to reduce energy consumption in agricultural WSNs while observing task constraints. The work considers only static networks and a single objective.

The second ACO-based approach was proposed by Zannou et al. [36]. Their approach seeks to minimize energy consumption by assigning the most capable nodes for a task and minimizing both the length and hop-counts of the transmission paths. Neither node failures nor mobility is considered, and only single-objective optimization is carried out.

In conclusion, metaheuristics are a very popular approach to solving the TAP for different objectives. However, very few works actually utilize full MOO. Overall, both node failures and mobility are considered in some of the works, but the purely static problem is still the most widely considered.

### 4.4. Cooperative and Antagonistic Methods

This section will analyze the works that fall into the category of cooperative and antagonistic methods based on game theory as described in Section 3.4, beginning with Consensus Algorithms.

Yin et al. [37] proposed a decentralized cooperative algorithm to tackle the TAP. For any given task, nodes negotiate the allocation until an agreement that satisfies all task requirements is met. The work considers only a static network, and while several metrics are evaluated, they are not optimized in a multi-objective fashion as the algorithm only considers node capabilities and their inclusion in past allocations when assigning new tasks. Task requests are propagated throughout the network in order to ensure that closer nodes have priority when reacting to requests. Furthermore, only independent tasks instead of dependent tasks are considered, as described in Section 2.

In [38], Pedroso et al. described their consensus-based algorithm to optimize the task execution delay in industrial IoT networks. By cooperatively pooling their capabilities, nodes reach a consensus on which to execute tasks. Tasks are clustered based on their similarity in terms of capability and neighborhood. Each cluster then seeks to build a consensus on a cluster leader, which accepts and assigns tasks for the cluster. Like the previous algorithm, this work only considers static networks, and performs only a single-objective optimization.

Mudassar et al. [39] proposed another decentralized, collaborative approach to increase reliability in IoT applications. Nodes are assigned to groups, which can independently operate without knowledge of the rest of the network. By coordinating with a group leader, the node groups can collaboratively execute tasks. If a node fails, a group can simply re-assign the task to nodes with free capacity without having to carry out re-optimization on the entire network. The work considers long-term failures due to resource exhaustion as well as short-term outages, resulting in full suitability according to the outlined criteria. Mobility was not considered at all, however, and no MOO was carried out, with nodes being selected purely based on available resources.

The next set of algorithms that belongs to this category are the auction-based algorithms described in Section 3.4.2. Edalat et al. [40] proposed a reverse-auction approach to maximize the network lifetime in resource-constrained WSN. To achieve energy balance, resource-constrained devices aim for the second-lowest-price bids. Cooperation is achieved by revealing all the private information of the nodes during the bidding process. If necessary, a low-energy node will use all of its available energy budget to complete an expensive task to ensure low task execution time. Evaluations were carried out to analyze the latency and energy consumption, but no multi-objective optimization was incorporated. The network considered was also mostly static, as low-energy nodes are set to sleep and not considered for task execution, avoiding failures during an allocation. As such, the algorithm is given a partial suitability ranking in terms of failures.

A non-cooperative auction-game approach was designed by Rahman et al. [41]. Nodes with similar capabilities are grouped into clusters, and cluster nodes bid on tasks to maximize their reward, which is based upon the expended energy of a task and gain of the executed task. Overall, the system aims to optimize the energy consumption of the system. The system model includes mobile nodes, and due to the clustering mechanism, node movement can be taken into account without full re-optimization, resulting in a partial suitability ranking. No failures are considered in this approach.

Joshi et al. [42] proposed a Stackelberg game approach to optimize the Latency in a IoT-based patient monitoring system. In the proposed approach, a cloud node acts as the leader and the IoT devices as followers. Neither mobility nor node failures are considered. A combined utility function includes both energy consumption and latency.

Overall, the cooperative and antagonistic game-theoretic approaches have to date mostly only been applied to the static TAP, and never in a MOO fashion. As such, the variety of metaheuristic approaches provides a wider spectrum for applications to dynamic networks and MOO.

### 4.5. Reinforcement Learning

In this section, algorithms belonging to the group of Reinforcement Learning (RL) algorithms described in Section 3.5 are analyzed.

Ding and Lin [43] proposed an approach to allocate dynamic tasks to a network of servers in order to minimize user costs by employing R-Learning [54]. While the tasks can dynamically change for this approach, the network model is static. As such, it is considered unfeasible with regard to mobility and node failures. Furthermore, only a single-objective optimization is performed.

An online RL approach for mobile IoT networks was developed by Yao and Ansari [44]. The objective of their approach is to minimize execution delay, e.g., latency, for tasks generated by mobile users. The RL is used to estimate the task costs and requirements based on previously completed tasks in order to find optimal allocations. While the users generating the tasks are considered mobile, the nodes in the network performing the tasks are static. No model for the prediction of user movement is included. As such, a partial feasibility for mobile networks is achieved. Node failures are omitted, and only optimization on a single objective is carried out. The same authors later proposed another, similar algorithm [45] suited for cost-efficient edge-cloud computing to enhance IoT services. However, the scenario changes little regarding the purpose of this analysis, and as such, provides similar ratings as their previous work, only degrading the mobility rating as no mobility is considered in the more recent work.

Zhou et al. [46] developed a privacy-preserving task allocation mechanism in massive crowd-sensing applications in cloud-enhanced IoT networks. The online learning algorithm is executed in the cloud, and estimates the reputation, or trustworthiness, of the other members in the network. Their approach can be combined with any of the centralized task allocation algorithms in order to incorporate privacy into the task allocation. As such, most of the criteria are inherited from the optimization algorithm employed. Thus, no rating is given for this work, but it is included in the table for sake of completeness.

Ma et al. [47] proposed a Deep Reinforcement Learning (DRL)-based approach to improve user experience and transmission stability in the vehicular IoT. Their system model includes both static, roadside nodes as well as fast-moving cars as part of the network. By caching data that will be required by vehicles in advance and in locations the vehicle will move through, the transmission time and efficiency can be improved. The system state is modeled as a Markov Decision Process, and DRL is used to obtain the optimal pre-caching and task allocations. As this approach includes models for the movement of the nodes, it is judged to be fully feasible for mobile scenarios as outlined by the criteria in Section 4.1. However, no node failures are considered, and the optimization process does not include multi-objective methodologies.

Li et al. [48] proposed a Graph Neural Network (GNN)-based approach to optimize the cruise control and task allocation of an IoT network incorporating UAVs. GNNs are an adaptation of Neural Networks (NNs) to graph-structured data and are a promising approach for future research concerning all network-related problems [55]. As the approach considers a moving UAV and plans its path to account for task allocations, it is deemed suitable in terms of mobility. However, no node failures were taken into account and only a single objective was considered.

With a few exceptions, the RL approaches were only applied to the static problem, and no approach incorporated a multi-objective design. However, RL and especially DRL approaches may be useful as part of other algorithms, such as surrogate models used for reducing the computational complexity [56] or the prediction of node dynamics, as shown by Weikert et al. [11]. GNNs especially are a promising approach to develop new solutions or enhance existing ones for the optimization problems of IoT networks.

### 4.6. Other Approaches

This section analyzes works employing methodologies that are not fitting to the approaches described in Section 3. As such, there will be a mix of different approaches.

In [49], Yu et al. proposed a Linear-Programming-based approach to optimize network lifetime in multi-hop WSNs. Linear Programming (LP) is a mathematical technique to solve problems wherein the objective function is linear. As such, they do not deal with Multi-Objective Optimization as described in Section 3.1. They then extend the approach by decomposing the problem, to improve reaction times in large networks and increase the feasibility for online optimization. While this approach guarantees optimal solutions, the computation time can be prohibitively large and full information is needed by the nodes responsible for calculating the solution. As such, network dynamics, which cannot be fully known by all nodes at all times, are not taken into account. In terms of metrics, only a single objective is optimized.

Another algorithm based on linear programming was developed by Xiaoyu et al. [50]. Their goal is to optimize task allocations in the vehicular IoT to optimize the maximum observed area with a minimum of participating vehicles. Their approach takes the trajectory of the vehicles into account to estimate their spatial and temporal availability for a given task. As such, it satisfies the requirements for full mobility feasibility as outlined in Section 4.1. Node failures are not considered, and only a single objective was optimized.

Gai et al. [51] proposed a heuristic algorithm based on hill-climbing to optimize the energy consumption while observing strict time constraints. Their approach uses a greedy approach as a baseline, and then slowly improves the solution by making small changes until a near-optimum is reached. No mechanism for dealing with node failures or node mobility are incorporated, and only energy consumption is optimized.

Stypsanelli et al. [52] performed an evaluation of several distinct task allocation procedures on a static networks. They measured the impact of these approaches on the response time, or latency, of the system. Their work suggests that even simple algorithms yield significant improvements over a static allocation. Unfortunately, the evaluation is limited to static networks and a single objective.

The final work categorized here was proposed by Alhaizaey et al. [53] and is based on Mixed-Integer Programming, which is an extension of Linear Programming including binary constraints. In the proposed approach, the binary variables are used to assign tasks to the nodes. The optimization goal is the overall latency of the system. The work only considered a small, static network.

Overall, these works were rarely applied to anything but the static network, and are not very well suited to multiple objectives. However, some of them can guarantee an optimal solution, which most of the works in the previous sections, such as metaheuristics, cannot.

## 5. Open Challenges

This section will address the open challenges that remain in this field, beginning with the criteria defined in Section 4.1 and concluding with some general challenges remaining in this field.

### 5.1. Node Failures

Out of the 37 reviewed works, only 4 are fully suitable for networks that include node failures. This is either achieved through retaining a large archive of previously optimized solutions [9,25], which has the drawbacks of increased computational effort to keep an optimized diverse archive as well as not guaranteeing an suitable solution in the archive, especially if there is a high rate of failure in the network. Introducing redundancy in the task allocations [26,39] is another option used to mitigate node failures, but comes at the cost of higher energy consumption and a high rate of failures can still cause task outages if all assigned nodes fail. Both approaches showed an improvement when faced with task failures in their respective works, but come with drawbacks regarding optimization quality or energy consumption. Effectively dealing with failures in a task allocation and optimizing the network throughout multiple failures to minimize the effect on other metrics as such remains a challenge for task allocation algorithms.

### 5.2. Node Mobility

Five of the reviewed works [9,11,47,48,50] incorporate a mechanism for preemptively planning the task allocations around the movement of nodes. However, two of these works assume either full knowledge of the trajectories [50] or actively plan the trajectories to improve the task allocation optimization [48]. To deal with the unknown future positions of nodes, only given the current node states, the remaining works all use a DRL-based approach in their optimization. This is either achieved by predicting future node positions [9,11] that are then used in the optimization process, or via directly optimizing based on the predicted reward of the network [47]. Notably, the works relying on predicted nodes can use any prediction mechanism in their algorithm and are not necessarily tied to a DRL approach. Nonetheless, incorrect predictions may greatly affect the performance of the algorithms. Furthermore, since these methods are based on evolutionary algorithms, a lot of time is required between the current state and any future prediction to allow for the completion of the optimization process. As such, scenarios with very fast nodes or generally erratic and highly dynamic systems still prove a significant challenge for task allocation optimization.

### 5.3. Multi Objective Optimization

None of the reviewed works provide an approach that optimizes all metrics described in Section 2.4, with most works only optimizing for one or two objectives. Furthermore, only five works used the concept of Pareto-Optimality [9,11,24,25,30], and four of these methods belong to the same family of algorithms. This shows a clear lack of MOO techniques in the current state-of-the-art, which generally relies on a weighted sum-approach to combine multiple objectives into one. This weighted-sum approach neglects the benefits of a posteriori MOO techniques, which can generate sets of non-dominated solutions and allow for the selection of an allocation fitting the current situation and requirements instead of relying on pre-defined weights of the optimization objectives.

### 5.4. Metrics

As previously noted, no approach actually optimizes for all metrics defined in Section 2.4, and none of the reviewed works evaluate their performance on all of the metrics. Evaluating for all metrics allows one to measure the impact of the optimization on the metrics, as improving performance in one metric may negatively impact another. This is another reason why MOO and Pareto-Optimality are such important concepts for future work in this field. Overall, latency and energy consumption are the most commonly evaluated metrics, with 16 and 14 of the reviewed works incorporating these metrics, respectively. While these are important objectives in the IoT landscape, an overt focus on a few metrics is a detriment to the versatility of IoT applications. The metrics of Availability and Reliability, which are especially critical for long-term stable operation, are currently under-represented with only five and four works, respectively, incorporating them into their evaluation. Current works also employ either computationally or resource-intensive methods to improve these metrics, which may be further improved by future research.

### 5.5. General Challenges

While some of the reviewed works consider either node failures or node mobility, only one work combined approaches to handle both. This approach is based upon Evolutionary Algorithms, which are a computationally expensive method as they require many evaluations of the objective functions and incur additional costs in order to calculate non-dominated solutions. While some work has been performed to reduce the complexity of the fitness evaluations by employing surrogate models [56], it remains a challenge to perform such algorithms with limited hardware resources. Another potential approach to combat this would be to distribute the optimization algorithm among the network, and thus sharing the computational load between many nodes. Evolutionary algorithms are suited for distributed optimization, although this comes with its own set of challenges and is an ongoing field of research [57].

Another open challenge for algorithms that rely on longer computation times are dynamic tasks that are not known ahead of time. While solutions can be quickly provided, this comes at a cost of their quality. Reinforcement-learning-based approaches [43,44,45] have been proposed to address the challenge of dynamic tasks, but only in a single-objective fashion and for mostly static networks. Networks with both dynamic movement and failures, as well as dynamically generated tasks, pose a significant open challenge in this field, as none of the individual problems can be deemed sufficiently solved. Especially EAs, one of the more common optimization techniques in this field, may be less suitable for dynamic tasks due to the computational effort required. Future work might include the combination of approaches, or the incorporation of transfer learning.

Large network or task graph sizes are also a relatively unexplored area in this field. With large numbers of nodes and tasks, or a high amount of objective functions, multi-objective optimization becomes increasingly difficult and computationally expensive. To optimize such networks, large-scale optimization techniques [58] are needed. Considering some notable IoT scenarios such as Smart Cities or the Internet of Vehicles, large-scale optimization may need to be incorporated into the task allocation process as the currently used methods are not sufficient.

Another important challenge is the difficulty to statistically compare different approaches in terms of their performance concerning the metrics defined in Section 2.4. This is due to the differences in the surveyed studies’ system models, assumptions, and methodology when evaluating, such as simulation-based evaluation vs. mathematical models. It is also not feasible to obtain a ground truth of optimal solutions for every possible scenario due to the NP-hardness of the TAP. Thus, a set of benchmark problems would be required to fully compare different approaches, implemented in one of the leading network simulation tools such as NS3 [59] or OMNeT++ [60]. For general MOO, many such benchmark problems [61] have been designed and, combined with open source implementations of many state-of-the-art algorithms [62,63], allow for the detailed analysis of different algorithms’ performances. Such a benchmark suite would greatly benefit future research in IoT Task Allocation.

## 6. Conclusions

In this paper, we defined the Task Allocation Problem for IoT networks, and divided it into distinct sub-problems based on network dynamics. We then performed an examination of the recent works to solve the Task Allocation Problem (TAP), and evaluated them regarding their suitability and incorporation of the distinct sub-problems. Additionally, we considered the inclusion of approaches capable of performing Multi-Objective Optimisation, as many real-world problems need to consider different goals that may be in conflict with one another. The approaches based on metaheuristics were the most diverse in terms of the inclusion of distinct sub-problems, and also the most commonly used type of algorithm, especially the subclass of EAs. The most suitable work regarding the specified criteria and providing MOO is also an EA-based approach. Algorithms based on Game Theory or Reinforcement Learning provide a less complete coverage of the problem, and seem more suited to a static approach, with limited applications to network dynamics. However, it is noted that reinforcement learning may be useful as a tool to improve the performance of other methodologies, either as a predictive model or as a surrogate for expensive fitness evaluations. Reinforcement learning has also been shown capable of dealing with dynamic tasks. Lastly, methods providing a guarantee for optimal solutions, such as Linear Programming-based algorithms, performed the worst in terms of the outlined criteria, as they are difficult to apply to dynamic problems, especially in MOO scenarios, but with guarantees regarding the focused objective. Overall, most current state of the art is still focused on fully or partially static problems. With greater inclusion of consumer products and smart devices into the IoT landscape, network dynamics will have a bigger impact on the performance of such optimizers. As such, further research into dealing with network dynamics without performing full re-optimization is necessary in the future. The aspect of reliability is only touched by some works and needs extended work to enable TAP algorithms to be usable in applications with safety requirements such as autonomous driving and vehicular IoT networks. Beyond this, establishing a benchmark suite to compare different methods against one another would be of great benefit for the research in this field, as there is currently a distinct lack of comparability between different approaches.

## Figures and Tables

**Table 1:** Full List of Node and Task Attributes.

Abbreviation	Attribute
$x_{i}^{N}$	Node position
$S_{i}^{N}$	Set of sensor/actuators of node
$p_{i}^{N}$	Node processing power
$m_{i}^{N}$	Node memory capacity
$l_{ij}^{N}$	Node transmission latency
$q_{i}^{N}$	Node energy
$p_{i}^{T}$	Task processing requirement
$m_{i}^{T}$	Task memory requirements
$C_{i}^{T}$	Set of task constraints
$X_{i}^{T}$	Task spatial constraints
$S_{i}^{T}$	Task sensor/actuator requirements
$c_{ij}^{T}$	Task communication cost

**Table 2:** Overview of state-of-the-art approaches.

Reference	Type	Failures	Mobility	MOO	Main Focus, Metrics
[19]	EA	o	−	−	Balance energy consumption, NL
[20]	EA	o	−	−	NL
[21]	EA	−	o	−	Measurement accuracy, E
[22]	EA	−	−	−	E
[23]	EA	o	−	o	A, L
[24]	EA	−	−	o	NL, L
[25]	EA	+	−	o	A, NL, L
[11]	EA	−	+	o	A, NL, L
[9]	EA	+	+	o	A, NL, L
[26]	PSO	+	−	o	Redundancy, E, L, R
[27]	PSO	−	−	o	NL, L, E
[28]	PSO	−	−	−	PSO/EA hybrid, L
[29]	PSO	−	−	o	L, E, R
[30]	PSO	−	−	o	Balanced energy consumption, L, E
[31]	PSO	−	−	−	E, R
[32]	PSO	−	−	−	Non−specified metrics
[33]	PSO	−	−	−	L
[34]	PSO	−	−	−	Execution cost, L, E
[35]	ACO	−	−	−	E
[36]	ACO	−	−	−	E
[37]	Consensus	−	−	−	task deadlines, E
[38]	Consensus	−	−	−	Allocation generation time
[39]	Consensus	+	−	−	Redundancy, R, A
[40]	Auction	o	−	−	NL
[41]	Auction	−	o	−	E
[42]	Stackelberg game	−	−	o	L
[43]	RL	−	−	−	Dynamic tasks, user cost
[44]	RL	−	o	−	Dynamic tasks, L
[45]	RL	−	−	−	Dynamic tasks, L
[46]	RL	n/a	n/a	n/a	Privacy measure for TAP
[47]	DL	−	+	−	Internet of Vehicles, data loss
[48]	GNN	−	+	−	Task miss ratio
[49]	LP,	−	−	−	NL
[50]	LP	−	+	−	Size of observable Area
[51]	Hill climbing	−	−	−	E, L
[52]	Multiple	−	−	−	Multiple
[53]	MIP	−	−	−	Allocation generation time, L

## Data Availability

Not applicable.

## Abbreviations

The following abbreviations are used in this manuscript:

*A*	Availability
*L*	Latency
*NL*	Network Lifetime
*R*	Reliability
$S_{i}$	Position Constraint
$c_{ij}$	Communication Cost
ACO	Ant-Colony-Optimization
DAG	Directed Acyclic Graph
DRL	Deep Reinforcement Learning
EA	Evolutionary Algorithm
EC	Energy Consumption
GNN	Graph Neural Network
IoT	Internet-of-Things
LP	Linear Programming
MOO	Multi-Objective Optimisation
MOP	Multi-Objective Problem
NN	Neural Network
PSO	Particle Swarm Optimization
RL	Reinforcement Learning
TAP	Task Allocation Problem
WSN	Wireless Sensor Network
